# Statistical analysis of a Bayesian classifier based on the expression of miRNAs

**DOI:** 10.1186/s12859-015-0715-9

**Published:** 2015-09-04

**Authors:** Leonardo Ricci, Valerio Del Vescovo, Chiara Cantaloni, Margherita Grasso, Mattia Barbareschi, Michela Alessandra Denti

**Affiliations:** 1 Department of Physics, University of Trento, Trento, I-38123 Italy; 2 Centre for Integrative Biology, University of Trento, Trento, I-38123 Italy; 3 Unit of Surgical Pathology, Trento, I-38122 Italy

**Keywords:** microRNA, Bayesian classifiers, Lung cancer, qRT-PCR gene expression measurement

## Abstract

**Background:**

During the last decade, many scientific works have concerned the possible use of miRNA levels as diagnostic and prognostic tools for different kinds of cancer. The development of reliable classifiers requires tackling several crucial aspects, some of which have been widely overlooked in the scientific literature: the distribution of the measured miRNA expressions and the statistical uncertainty that affects the parameters that characterize a classifier. In this paper, these topics are analysed in detail by discussing a model problem, i.e. the development of a Bayesian classifier that, on the basis of the expression of miR-205, miR-21 and snRNA U6, discriminates samples into two classes of pulmonary tumors: adenocarcinomas and squamous cell carcinomas.

**Results:**

We proved that the variance of miRNA expression triplicates is well described by a normal distribution and that triplicate averages also follow normal distributions. We provide a method to enhance a classifiers’ performance by exploiting the correlations between the class-discriminating miRNA and the expression of an additional normalized miRNA.

**Conclusions:**

By exploiting the normal behavior of triplicate variances and averages, invalid samples (outliers) can be identified by checking their variability via chi-square test or their displacement by the respective population mean via Student’s t-test. Finally, the normal behavior allows to optimally set the Bayesian classifier and to determine its performance and the related uncertainty.

**Electronic supplementary material:**

The online version of this article (doi:10.1186/s12859-015-0715-9) contains supplementary material, which is available to authorized users.

## Background

MicroRNAs (miRNAs or miRs) are small non-coding single-stranded RNAs, 19–25 nucleotides in length, acting as negative regulators of gene expression at the post-transcriptional level. More than 1000 miRNAs are transcribed from miRNA genes in the human genome. A single miRNA is able to modulate hundreds of downstream genes by recognizing complementary sequences in the 3^′^ untranslated regions (UTRs) of their target messenger RNAs. It has been estimated that in humans about 30 % of messenger RNAs are under miRNA regulation. The biological functions of miRNAs are diverse and include several key cellular processes, such as differentiation, proliferation, cellular development, cell death and metabolism.

In the last decade, evidences have accumulated to indicate that miRNAs play a role in the onset and progression of several human cancers [1]. The transcription or processing of some miRNAs is altered in neoplastic tissues, with respect to their normal counterparts. miRNAs whose levels increase in tumors are referred to as oncogenic miRNAs (onco-miRs), sometimes even if there is no evidence for their causative role in tumorigenesis. On the other hand, miRNAs down-regulated in cancer are considered tumor suppressors.

In parallel to these studies, the effectiveness of miRNAs as markers for tracing the tissue of origin of cancers of unknown primary origin was demonstrated by several authors, and the utility of miRNAs levels as diagnostic and prognostic markers became clear (reviewed by [2]). The main advantage of the use of miRNAs as markers resides in the ease of their detection and in their extreme specificity. miRNAs are stable molecules well preserved in formalin fixed, paraffin embedded tissues (FFPE) as well as in fresh snap-frozen specimens, unlike larger RNA molecules as messenger RNAs [3]. The finding that miRNAs have an exceptional stability in several tissues suggested that these tiny molecules were also preserved, detectable and quantifiable in plasma and in other biofluids, such as urine, saliva, cerebro-spinal fluid and amniotic fluid [4]. Circulating miRNAs are attracting attention as markers not only for cancer but also for neurodegenerative diseases (reviewed by [5]) as they have some important features: non-invasivity, specificity, early detection, sensitivity and ease of translatability from model systems to humans.

Methods based on next generation sequencing (NGS), microarray and quantitative reverse-transcription polymerase chain reaction (qRT-PCR) are currently used for miRNA profiling and for the identification of miRNAs differently expressed in tumor samples and in matched, healthy tissue. The majority of miRNA profiling studies have been so far carried out by using microarrays. These studies have provided signatures consisting in few to several (5–30) distinct miRNAs [6]. However, the large amount of data obtained by microarray and NGS profiling needs to be transposed into clinical trials by developing an easily performed, cost-effective and serviceable assay that can analyze the cancer-specific miRNAs for cancer diagnosis and prognosis. Such an analysis has been so far relying on qRT-PCR assays, performed by measuring the levels of a restricted number of miRNAs (see review by [7]).

The realization of classifiers based on the expression of miRNAs is widely discussed in the scientific literature. Within the context of lung cancers (see, for example, [4, 8–12]) the work by [13] describes a classifier that distinguishes squamous from nonsquamous non-small-cell lung carcinomas, by using miR-205 as a specific marker and miR-21, snRNA (small nuclear RNA) U6 as normalizers. The approach followed is essentially machine learning: the classifier relies on a sample score and a threshold. A more elaborated support vector machine, which uses the combination of 5 miRNAs for lung squamous cell carcinoma diagnosis, is described in [14]. A receiver operating characteristic curve analysis to evaluate the possibility of diagnosing the histologic subtype of pulmonary neuroendocrine tumors via altered expression of miR-21, miR-155, let-7a is discussed in [15] (a similar statistical approach is described in [16]).

These classifiers are generally declared to be efficient. For example, [13] report a sensitivity of 96 % and a specificity of 90 %. However, at least two aspects are widely overlooked in the scientific literature: first, the distribution of the measured miRNA expressions; second, the statistical uncertainty that unavoidably affects the parameters that characterize a classifier and its performance. Both aspects are crucial in order to assess the reproducibility, and thus the reliability, of a classifier. The goal of the present paper is to close these gaps. Our analysis concerns a Bayesian classifier based on the expression of a single class-discriminating miRNA, with additional miRNAs that are used either as normalizers or as performance-enhancer via noise-reduction.

In the present paper the following issues are discussed: normal distribution of the triplicate variance and identification of outliers; improvement of accuracy via normalizers; class-discriminating measures and their distributions; identification of “bias” outliers; assessment of a classifier’s performance; finally, improvement of a classifier’s performance by exploiting correlations.

As a prototypical case we discuss throughout the paper the development of a classifier that assigns samples either to adenocarcinomas (ADC) or to squamous cell carcinomas (SQC). The two classes ADC, SQC are henceforth referred to as the *target* and the *versus* class, respectively. The miRNAs used are miR-205, miR-21 and snRNA U6.

## Methods

### Distribution of triplicates: normally-distributed variance and outlier identification

Given a sample stemming from a patient, a set of miRNAs is measured in triplicate by using qRT-PCR. For each miRNA, the sample mean *x* of the corresponding triplicate and the related sample standard deviation *s* are calculated. To provide an *a priori* knowledge on the samples, each one was classified via immunohistochemical analysis and gene profiling into one of different categories of lung tumors. We use data based on lung carcinoma biopsies retrieved from the archives of the Unit of Surgical Pathology of the S. Chiara Hospital in Trento, Italy. The research project had been approved by the Ethical Committee of the Trentino Public Health System (Azienda Provinciale per i Servizi Sanitari). Most of the data analyzed here were previously published by our research group [17]. All datasets are available as Supplementary material in the Additional file 1.

For each miRNA, the distribution of the variances *s* ^2^ can be described by a normal chi-square distribution with number of degrees of freedom *ν* equal to 2: ${s^{2} / \sigma ^{2} \sim \chi ^{2}_{\nu =2}}$. To prove this behavior, we fitted the cumulative distribution ${F\left (\chi ^{2}_{\nu =2};\,\nu =2\right)}$ of the chi-square with *ν*=2 to the cumulative distribution of the measured variances: knowing that *F*(*s* ^2^/*σ* ^2^; 2)=1− exp[−*s* ^2^/(2*σ* ^2^)], the fit was carried out by searching the population variance *σ* ^2^ that minimizes the Kolmogorov-Smirnov (K-S) statistic *D*. We used the K-S test because it is less sensitive to outliers than other statistical tests and does not require any assumption on the distributions. On the contrary, for example, the estimation of the population variance *σ* ^2^ out of the set of sample variances is valid only if the sample distribution is already known to be normal. The *p*-values resulting from the fit (see Table 1) show that the variance distributions are indeed compatible with chi-square distributions.

**Table 1:** Statistics of the standard deviation for the sets of triplicates of miR-205, miR-21 and snRNA U6, as well as for the snRNA U6 set devoid of outliers

miRNA	Set size	*σ*	Fit’s *p*-value	*σ*_max_	〈*s* ^2^〉^1/2^	[ 〈*s* ^4^〉−〈*s* ^2^〉^2^]^1/4^
miR-205	37	0.25	0.60	**0.61**	0.26	0.25
miR-21	39	0.19	0.99	**0.46**	0.20	0.21
snRNA U6	39	0.19	0.10	**0.48**	0.26	0.30
snRNA U6	35	0.17	0.13	-	0.20	0.21
without outliers						

The population variance *σ* results from the Kolmogorov-Smirnov test analysis; the related *p*-value is reported. The threshold *σ*_max_ (bold), above which a value is deemed to be an outlier, is set to 2.448·*σ*. The two rightmost columns provide 〈*s* ^2^〉^1/2^ and [ 〈*s* ^4^〉−〈*s* ^2^〉^2^]^1/4^ (see main text)

As a result, an outlier can be identified by checking its variability via the chi-square test: we assume a triplicate of a given miRNA to be an outlier if its sample standard deviation *s* exceeds the critical value *σ*_max_ corresponding to the significance level *α*=0.05. The critical value *σ*_max_ is given by [−2 log*α*]^1/2^ *σ*≃2.448 *σ*, where *σ* is the population standard deviation assessed for that miRNA. The *σ*_max_ values are reported in Table 1. The outlier definition used here implies that the significance level *α* corresponds to the rate of statistical false alarms (type I errors), i.e. the rate of valid triplicates that are falsely deemed to be outliers.^1^

According to this procedure, the triplicate sets of both miR-205 and miR-21 contained no outliers, whereas the snRNA U6 contained 4 outliers. These samples were excluded from the following analysis. Table 1 also shows the statistics of the standard deviation for the snRNA U6 data set devoid of outliers.

We note that, for snRNA U6, the two values of *σ* are very similar. This fact reflects, as mentioned above, the robustness to outliers of the K-S approach. In addition, for all data sets devoid of outliers, the population standard deviation *σ* is approximately equal to both the root-mean-square sample standard deviation 〈*s* ^2^〉^1/2^ and the fourth root of the variance of variances [〈*s* ^4^〉−〈*s* ^2^〉^2^]^1/4^. Because *ν*=2, this behavior provides further evidence to the null hypothesis that samples are drawn from the same normal distribution.

For each sample, i.e. patient, of the set devoid of outliers, we henceforth use the following notation for the sample mean *x* of the available triplicates: *x*_U6_ for the snRNA U6 triplicate, *x*_21_ for the miR-21 triplicate, and *x*_205_ for the miR-205 triplicate. In addition, *x*_205_, *x*_21_, *x*_U6_ will be referred to as *measures*.

### Distribution of triplicates: accuracy and normalization

A main issue to cope with towards the development of a reliable classifier is accuracy. The question is whether the values of the sample means of the triplicates are constant over different experimental sessions – i.e. measurements taken at different times and/or with different set-ups – or, rather, have to be *normalized* in order to remove experimental bias.

Table 2 shows the results of a statistical analysis, in terms of sample mean $\overline {X}$ and sample standard deviation *S*, carried out for *x*_21_, *x*_U6_ and their difference *x*_21_−*x*_U6_ on data gathered in two different experimental sessions. For both single-miRNA values *x*_21_ and *x*_U6_, and both for the target and the versus class, the sample means significantly differ between the two sessions. However, this is not the case for the sample means of *x*_21_−*x*_U6_: the difference of the sample mean of *x*_21_−*x*_U6_ between session II and session I is 0.4±0.4 for the target class, and −0.2±0.8 for the versus class; in both cases the difference is less than twice the respective sample standard deviation (*p*>0.05). Remarkably, the sample standard deviations, and consequently the uncertainties on the sample means, do not significantly differ between the two sessions.

**Table 2:** Statistics of the measures *x*_21_, *x*_U6_, *x*_21_−*x*_U6_ obtained on data stemming from two different experimental sessions

Session	Measure	Class	Set size	$\overline {X}$	*S*
I	*x*_21_	target	21	18.4(2)	1.0(2)
		versus	18	19.2(6)	2.6(5)
	*x*_U6_	target	19	25.0(3)	1.4(2)
		versus	16	25.8(5)	1.9(3)
	*x*_21_−*x*_U6_	target	19	–6.5(3)	1.4(2)
		versus	16	–6.6(6)	2.4(4)
II	*x*_21_	target	19	21.4(3)	1.1(2)
		versus	17	21.9(6)	2.3(4)
	*x*_U6_	target	20	27.4(3)	1.2(2)
		versus	16	28.4(6)	2.6(5)
	*x*_21_−*x*_U6_	target	17	–6.1(3)	1.1(2)
		versus	16	–6.8(6)	2.5(5)

For each session, measure and class, the two symbols $\overline {X}$ and *S* correspond to sample mean and sample standard deviation of the *x* values, respectively

The necessity to improve accuracy by suitably normalizing an oncomir has been extensively discussed in the scientific literature (see for example [18]). Thus, in accordance with many previous works, we use snRNA U6 to normalize *x*_21_ and *x*_205_. The following notation is henceforth used: *Δ* *x*_205_≡*x*_205_−*x*_U6_; *Δ* *x*_21_≡*x*_21_−*x*_U6_.

### MiRNA statistics

In this section, the statistics of *Δ* *x*_205_, *Δ* *x*_21_, snRNA U6 is discussed.

**Fig. 1:**
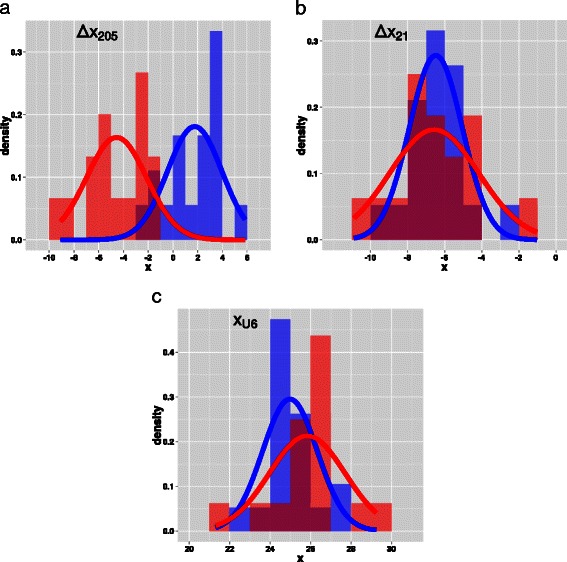
Histograms of *Δ* *x*_205_ (top, left), *Δ* *x*_21_ (top, right), *x*_U6_ (bottom) for samples belonging to the target class *ADC* (blue) and to the versus class *SQC* (red). Overlapping regions are in magenta. The bin width is equal to 1. Means and standard deviations of the Gaussian curves that fit the data are reported in Table 3. Each histogram is normalized to the respective set size

As stated above, each sample was classified into one of the two classes *ADC* and *SQC* via immunohistochemical analysis and gene profiling. Figure 1 shows the histograms of *Δ* *x*_205_, *Δ* *x*_21_, *x*_U6_ for samples belonging either to the target class *ADC* or to the versus class *SQC*.

**Table 3:** Statistics of *Δ* *x*_205_, *Δ* *x*_21_, *x*_U6_, *y*_*DV*_ (see Eq. (2)), *y*_*opt*_ (see Eq. (10))

Measure	Class	Set size	$\overline {X}$	*S*	TN	t-test
*Δ* *x*_205_	target	18	1.7(5)	2.2(4)	0.71	2·10^−8^
	versus	15	–4.6(6)	2.4(5)	0.26	
*Δ* *x*_21_	target	19	–6.5(3)	1.4(2)	0.53	0.90
	versus	16	–6.6(6)	2.4(4)	0.94	
*x*_U6_	target	19	25.0(3)	1.4(2)	0.40	0.14
	versus	16	25.8(5)	1.9(3)	0.25	
*y*_*DV*_	target	18	4.9(5)	2.0(3)	0.96	5.5·10^−11^
	versus	15	–1.3(4)	1.7(3)	0.98	
*y*_*opt*_	target	18	6.9(5)	1.9(3)	0.78	2.6·10^−11^
	versus	15	0.7(4)	1.6(3)	0.24	

The digits in parentheses correspond to the uncertainty on the respective least significant digits. The column marked with TN (test of normality) contains the *p*-values yielded by the Shapiro-Wilk test to check whether the data contained in a histogram are consistent with a normally distributed parent population. Finally, the rightmost column reports the *p*-value of Student’s t-test to check the null hypothesis that the target and versus sets have the same population mean; to this purpose, the variance is estimated separately for each group and the Welch modification to the number of degrees of freedom is used

By means of the Shapiro-Wilk test of normality, each histogram was shown to be consistent with a normal parent population. Consequently, we assume that, for each of the two classes, the measures *Δ* *x*_205_, *Δ* *x*_21_, *x*_U6_ are normally distributed with a mean and a standard deviation that are respectively estimated by the sample mean $\overline {X}$ and the sample standard deviation *S* of the *x* values (triplicates). The results are reported in Table 3. The proof that the measures of interest are compatible with normal distributions makes up a crucial step towards the optimization of the Bayesian classifier and the determination of its performance, inclusively of the related uncertainty (see below).

With regard to *Δ* *x*_205_, the histograms of the target class *ADC* and the versus class *SQC* are well-separated: Student’s t-test provides *p*<10^−7^ (see Table 3). Conversely, both for *Δ* *x*_21_ and *x*_U6_, the overlapping of the histograms of the two classes is confirmed by Student’s t-test, which provides *p*=0.90 and *p*=0.14, respectively. Therefore, only the measure *Δ* *x*_205_ is a good candidate to classify samples into *ADC* or *SQC*.

### A Bayesian classifier

To develop a classifier, any linear combination *y* of the available measures can be used. Given a linear combination *y* of the measures *Δ* *x*_205_, *Δ* *x*_21_, *x*_U6_, we assume the following classification rule to hold:

(1) $$ \text{classifier output class} =\!\! \left\{\!\!\begin{array}{ll} SQC\text{(versus class)} & \!\text{if}\; y < \chi \, \,, \\ ADC\text{(target class)} & \!\text{if}\; y \geqslant \chi \, \,, \end{array}\right.   $$

where *χ* is a fixed threshold. For example, in the works by [13, 17] the linear combination

(2) $$ y_{DV} = \Delta x_{205} - 0.5 \cdot \Delta x_{21}  $$

was used and a threshold *χ*=2.5 was set.

The discriminator approach described in Eq. (1) requires tackling three main issues: finding a suitable linear combination *y*; finding a suitable value for *χ*; analyzing the performance of the classifier.

Given a linear combination *y* (that may also coincide with one of the three measures), the threshold *χ* can be determined by calculating the value that, according to Eq. (1), maximizes the accuracy (or rate of correct responses) *p*_*c*_:

(3) $$ p_{c} = p_{T} H + p_{V} C \,.  $$

In this equation, *p*_*T*_ and *p*_*V*_ correspond to the prior presentation probabilities of the target class and the versus class, respectively, whereas *H* and *C* respectively correspond to the sensitivity and the specificity of the classifier, provided that the *condition positive* is taken to correspond to the target class [19]. Under the assumption that the values of *y* are normally distributed, sensitivity and specificity are given by the following expressions:

(4a) $$\begin{array}{*{20}l}  H &= \Phi\left(\frac{\mu_{T} - \chi}{\sigma_{T}} \right) \,, \end{array} $$

(4b) $$\begin{array}{*{20}l} C &= \Phi\left(\frac{\chi - \mu_{V}}{\sigma_{V}} \right) \,, \end{array} $$

where *Φ*(*x*) is the standard normal cumulative distribution. The optimal position of *χ* is given by one of the roots (the most appropriate one!) of the following second-degree equation:

(5) $$ \eta \chi^{2} - 2 \beta \chi + \gamma - 2 \log \frac{p_{T}}{p_{V}} = 0 \,,   $$

where

(6a) $$\begin{array}{*{20}l} \eta &= \frac{1}{{\sigma_{T}^{2}}} - \frac{1}{{\sigma_{V}^{2}}} \,, \end{array} $$

(6b) $$\begin{array}{*{20}l} \beta &= \frac{\mu_{T}}{{\sigma_{T}^{2}}} - \frac{\mu_{V}}{{\sigma_{V}^{2}}} \,, \end{array} $$

(6c) $$\begin{array}{*{20}l} \gamma &= \frac{{\mu_{T}^{2}}}{{\sigma_{T}^{2}}} - \frac{{\mu_{V}^{2}}}{{\sigma_{V}^{2}}} + 2 \log \frac{\sigma_{T}}{\sigma_{V}} \,. \end{array} $$

The uncertainties on the three coefficients *η*, *β*, *γ*, and thus on the threshold *χ*, are computed by means of standard error propagation. We remind that *μ*_*T*_, *σ*_*T*_, *μ*_*V*_, *σ*_*V*_ are evaluated as sample means and sample standard deviations, and are therefore uncertainty-affected: for example, the errors on *μ*_*T*_, *σ*_*T*_ are ${\sigma _{T}/\sqrt {N_{T}}}$, ${\sigma _{T}/\sqrt {2(N_{T}-1)}}$, respectively, where *N*_*T*_ is the number of triplicates belonging to the target class.

The threshold *χ* depends on the ratio *p*_*T*_/*p*_*V*_ of the prior occurrence probabilities of the two classes of tumors. These probabilities are typically inferred from epidemiological studies. If prior probabilities are balanced, the ratio *p*_*T*_/*p*_*V*_ is unitary and the related term in Eq. (5) vanishes. Table 4 reports the values of *χ* and the respective uncertainties for each of the measures dealt with in this paper, in the case of balanced prior probabilities. For *x*_U6_ the normal curve for the target class lays on the left of the normal curve for the versus class (i.e., ${\overline {X}_{\textit {ADC}} < \overline {X}_{\textit {SQC}}}$; see Fig. 1 and Table 3). So, in the case of this measure, the application of the rule of Eq. (1) requires the linear combination *y* being set to −*x*_U6_.

**Table 4:** Thresholds *χ*_10:90_, *χ*, *χ*_90:10_. For each measure, the thresholds were evaluated via Eqs. (5, 7) by assuming balanced prior probabilities

Measure	*χ*_10:90_	*χ*	*χ*_90:10_
*Δ* *x*_205_	–3.2(7)	–1.3(4)	0.6(8)
*Δ* *x*_21_	–10(1)	–8.2(6)	–6.4(6)
−*x*_U6_	–29(1)	–26.1(5)	–24(1)
*y*_*DV*_	0.5(5)	1.7(4)	2.8(6)
*y*_*opt*_	2.4(5)	3.6(4)	4.6(6)

The digits in parentheses correspond to the uncertainty on the respective least significant digits

### Estimated classifier performance

A new sample, i.e. a new value of the measure *y* whose costituent triplicates were checked not to be outliers, can now be assigned to the target or the versus class according to the rule of Eq. (1). An estimate of the reliability of this assignment is provided by Bayes’ theorem: the odds that the new sample, given its *y*, belongs to the target class are given by the likelihood ratio

(7) $$ \frac{P_{T}}{P_{V}} = \exp \left[ -\frac{1}{2} \left(\eta y^{2} - 2 \beta y + \gamma - 2 \log \frac{p_{T}}{p_{V}} \right) \right] \,,  $$

where *p*_*T*_ and *p*_*V*_ are the two prior probabilities (*p*_*T*_+*p*_*V*_=1), and *η*, *β*, *γ* are given by Eq. (6). From Eq. (5) it follows that the odds at *y*=*χ* are 50:50. By means of Eq. (7), the thresholds *χ*_10:90_, *χ*_90:10_ for the odds 10:90 and 90:10 can be determined. The values of these thresholds in the case of balanced prior probabilities are reported in Table 4.

### Bias outliers

In the previous sections, we have addressed the issue of outlier identification by using the triplicate variability. The normality of the “target” and “versus” distributions of the measure of interest *y* allows for the identification of a second kind of outliers: given a value *y*, one can promptly evaluate – via Student’s t distribution – the one-tailed probability of obtaining a more extreme value, i.e. a value more displaced by the population mean than the value *y*. If such *p*-value is less than a given significance level (for example, 1 %), the value *y* can be deemed to be a “bias” outlier, i.e. an outlier due to a bias in the triplicate estimates.

### Improvement of a classifier’s performance

Looking at Student’s t statistic provides two possible strategies to improve the performance of a classifier. First, one can enhance the difference at the numerator of Student’s t, namely *μ*_*T*_−*μ*_*V*_; this solution requires the linear combination of the available class-discriminating measures (in the present case *Δ* *x*_205_) with new, additional measures that also reliably discriminate between the two classes. Such linear combination has to be optimized by means of methods like, for example, principal component analysis or support vector machines. The discussion of these methods goes beyond the goals of the present paper. The second strategy to improve the performance of a classifier consists in reducing the denominator in the expression of Student’s t by linearly combining available measures with new ones. These new measures are not required to be class-discriminating. In the following section the second strategy is analyzed in detail.

### Analysis of correlation

Let *y*_*a*_, *y*_*b*_ be two measures: *y*_*a*_ is supposed to discriminate between two classes (according to Student’s t-test), whereas *y*_*b*_ is not. In the present paper, we can have *y*_*a*_=*Δ* *x*_205_ and *y*_*b*_=*Δ* *x*_21_ or *y*_*b*_=*x*_U6_. Let *y* be a linear combination of *y*_*a*_, *y*_*b*_ as follows:

(8) $$ y = y_{a} + c y_{b} \,,  $$

where *c* is a coefficient to be determined. Taking the average of the last equation provides

$$\mu = \mu_{a} + c \mu_{b} \,. $$

Because measure *y*_*b*_ does not discriminate between the two classes, we have *μ*_*b*,*T*_≈*μ*_*b*,*V*_ (the means of the distributions of *y*_*b*_ for the target and the versus class are not significantly different). Consequently, *μ*_*T*_−*μ*_*V*_≈*μ*_*a*,*T*_−*μ*_*a*,*V*_, i.e. nothing significant can be expected with regard to the numerator of Student’s t statistic when *μ*_*a*_ is replaced by *μ*.

With regard to standard deviations, from Eq. (8) it follows:

$$\sigma^{2} \simeq {\sigma_{a}^{2}} + c^{2} {\sigma_{b}^{2}} + 2 c r \sigma_{a} \sigma_{b} \,, $$

where *r* is the linear correlation coefficient between *y*_*a*_ and *y*_*b*_. If *r*=0, adding the new measure *y*_*b*_ is definitely detrimental for the sake of discrimination because the variance is increased by a term ${c^{2} {\sigma _{b}^{2}}}$. However, in the case of a significant correlation, setting

(9) $$ c \simeq - r \frac{\sigma_{a}} {\sigma_{b}} \,,  $$

reduces the standard deviation as follows:

$$\sigma \simeq \left(1 - r^{2} \right)^{1/2} \sigma_{a} \,. $$

Consequently, the additional measure *y*_*b*_ can be deemed to be a sort of noise-reducer for the class-discriminating measure *y*_*a*_.

The previous argument still holds if, rather than regarding the whole data set, the correlation appears only on the data subset corresponding to one of the two classes. Therefore, if *c* is set according to Eq. (9), one of the two standard deviations *σ*_*T*_, *σ*_*V*_ is reduced whereas the other is enhanced. The net result can be still an increase of the classifier’s performance. The optimal value of *c* can be assessed by standard analytical and numerical techniques.

## Results

### A classifier for ADC vs. SQC

**Fig. 2:**
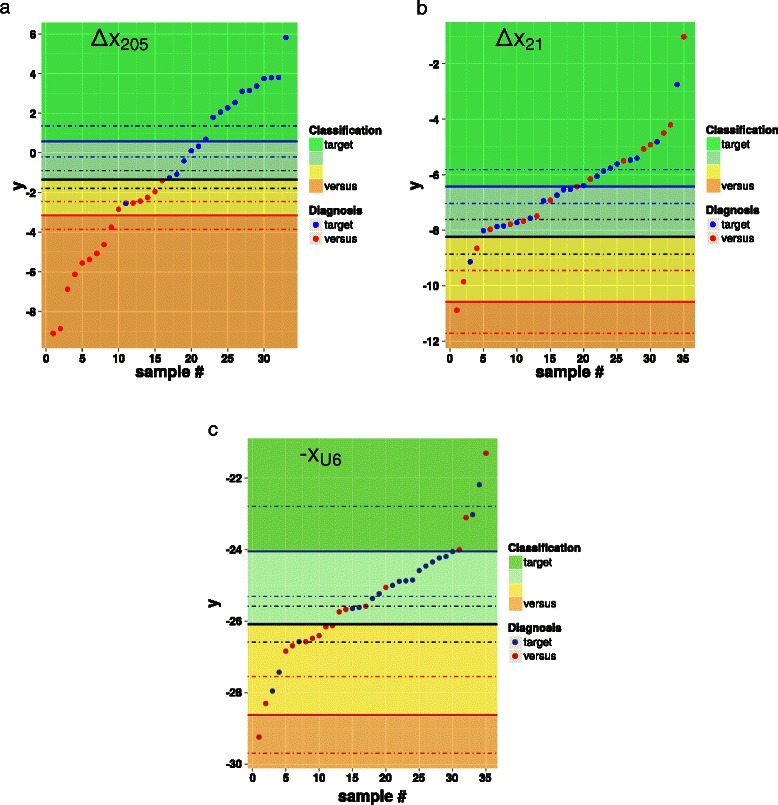
Scatter plots of *Δ* *x*_205_ (top, left), *Δ* *x*_21_ (top, right), −*x*_U6_ (bottom). Dots corresponding to samples of the target class *ADC* and the versus class *SQC* are colored in blue and red, respectively. In each plot, the black, bold line represents *χ*, wheres the two black, dashed lines correspond to *χ*±*d* *χ*. Similarly, the three red lines and the three blue lines represent *χ*_10,90_, *χ*_10,90_±*d* *χ*_10,90_ and *χ*_90,10_, *χ*_90,10_±*d* *χ*_90,10_, respectively (see Table 4)

For each of the three measures of interest, Fig. 2 shows the scatter plot of the respective values as well as the three thresholds *χ*_10:90_, *χ*, *χ*_90:10_. The dot color corresponds to the class the sample was assigned to via immunohistochemical analysis and gene profiling (diagnosis). The plots contains four different regions, bounded by the three thresholds and corresponding to different outcomes of the classifier: orange ⇒ versus class with odds larger than 90:10; yellow ⇒ versus class with odds between 50:50 and 90:10; light green ⇒ target class with odds between 50:50 and 90:10; green ⇒ target class with odds larger than 90:10.

The reliability of the classifiers based on each of the three available measures can be inferred by considering the *confusion matrix* reported in Table 5. The accuracy is equal to 97 % for *Δ* *x*_205_, 60 % for *Δ* *x*_21_, and 71 % for −*x*_U6_. Due to overfitting, the performance of the classifiers based on the last two measures are apparently satisfactory, though non competitive with that of the classifier based on *Δ* *x*_205_. However, if only high-reliability responses are considered, namely those with odds at least 90:10, the accuracies drop to 64 %, 29 %, 9 %, respectively: while the accuracy of the classifier based on *Δ* *x*_205_ is still satisfactory, the other two measures do not provide reliable outcomes. This behavior is linked to the t-statistic regarding the separation of the distributions corresponding to the two classes with respect to the widths of the distributions (see Fig. 1).

**Table 5:** Confusion matrix for classifiers of *ADC* vs. *SQC* relying on *Δ* *x*_205_, *Δ* *x*_21_, *x*_U6_ as well as on *y*_*DV*_ (see Eq. (2)) and *y*_*opt*_ (see Eq. (10))

		Classification			
Measure	Diagnosis	Target		Versus	
		*ρ*>9	9>*ρ*>1	9>*ρ*>1	*ρ*>9
*Δ* *x*_205_	target	**12**	5	*1*	***0***
	versus	***0***	*0*	6	**9**
*Δ* *x*_21_	target	**9**	9	*1*	***0***
	versus	***8***	*5*	2	**1**
−*x*_U6_	target	**2**	14	*3*	***0***
	versus	***3***	*4*	8	**1**
*y*_*DV*_	target	**16**	1	*1*	***0***
	versus	***0***	*1*	1	**13**
*y*_*opt*_	target	**16**	1	*1*	***0***
	versus	***1***	*0*	0	**14**

The quantity *ρ* corresponds to the odds: *ρ*=1⇔ 50:50; *ρ*=9⇔ 90:10. Entries in italic refer to false responses (false positives and negatives); the other entries refer to correct responses (true positives and negatives); high-reliability entries, with odds at least 90:10, are marked in bold

In the case of the classifier based on *Δ* *x*_205_, by relying on Eqs. (3, 4) a maximum accuracy of 91.4 *%* ± 3.9 *%* can be predicted.

### Improved classifier for ADC vs. SQC

Table 6 reports the correlation coefficient *r* for the pair (*x*_205_, *x*_U6_), i.e. directly between miR-205 and snRNA U6, and for the pair (*Δ* *x*_205_, *Δ* *x*_21_).

**Table 6:** Pearson correlation coefficient *r* for the pairs (*Δ* *x*_205_, *Δ* *x*_21_), (*x*_205_, *x*_U6_)

Correlation pair	Set	Size	*r*	*p*-value
	overall	33	-0.02	0.92
(*x*_205_, *x*_U6_)	target	18	0.13	0.60
	versus	15	0.43	0.11
	overall	33	0.40	***0.022***
(*Δ* *x*_205_, *Δ* *x*_21_)	target	18	0.49	***0.04***
	versus	15	0.75	***0.0012***

The *p*-value refers to the null hypothesis that the data from the two pair elements are uncorrelated. Significant *p*-values (<0.05) are marked in bold

We first note that no correlation between miR-205 and snRNA U6 can be significantly inferred. Consequently, despite being a normalizing miRNA – i.e. a bias-reducer– for miR-205, snRNA U6 is useless as a noise-reducer for miR-205. On the contrary, the class-discriminating measure *Δ* *x*_205_ has a significant correlation with *Δ* *x*_21_, which can be therefore used to improve the classification performance of *Δ* *x*_205_. Given an overall correlation coefficient *r* of 0.022, Eq. (9) provides a value of *c* approximately equal to –0.8 so that the optimal linear combination *y*_*opt*_ is:

(10) $$ y_{opt} = \Delta x_{205} - 0.8 \cdot \Delta x_{21} \,.  $$

Tables 3, 4 and 5 report the statistics of the measure *y*_*opt*_, and the thresholds and confusion matrix of the classifier relying on this measure, respectively. For the sake of comparison, the same tables also show the data of the classifier relying on the measure *y*_*DV*_ defined in Eq. (2), which was the topic of previous works [13, 17].

**Fig. 3:**
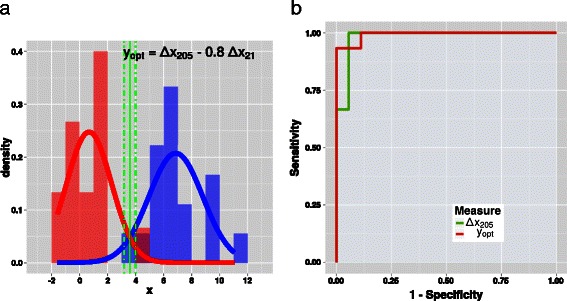
Histograms (left) of *y*_*opt*_=*Δ* *x*_205_−0.8·*Δ* *x*_21_ for samples belonging to the target class *ADC* (blue) and to the versus class *SQC* (red). Overlapping regions are in magenta. The bin width is equal to 1. Each histogram is normalized to the respective set size. The green bold line represents the discrimination threshold *χ*=3.6, whereas the green dashed lines represent the threshold displaced by its uncertainty, i.e. *χ*±*d* *χ*, with *d* *χ*=0.4 (see Table 4). ROC curves (right) of the classifier based on *Δ* *x*_205_ (green line) and of the classifier based on *y*_*opt*_ (red line) [20]. The increase of the AUC (area under the curve) from 0.9815 to 0.9926, respectively, is another marker of the improvement of the classifier

Testing the same-parent-distribution null hypothesis via Student’s t statistic provides *p*=2.6·10^−11^, half of the value obtained by testing t on the histograms generated by using the linear combination *y*_*DV*_. Figure 3 shows the histograms of *y*_*opt*_ for samples belonging either to the target class *ADC* or to the versus class *SQC*. The Shapiro-Wilk test of normality yielded *p*-values of 0.78 (target class) and 0.24 (versus class).

**Fig. 4:**
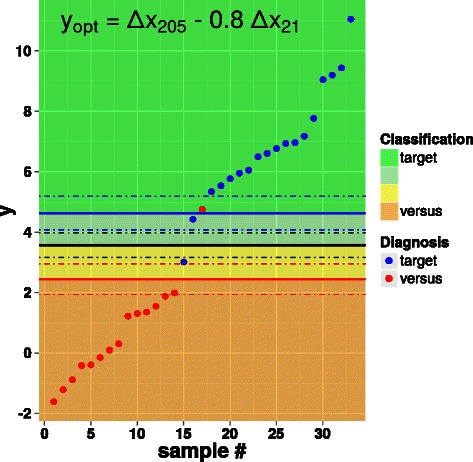
Scatter plot of *y*_*opt*_=*Δ* *x*_205_−0.8·*Δ* *x*_21_. See Section “A classifier for ADC vs. SQC” and the caption of Fig. 2 for the color code of dots, lines and shaded areas. The values of the thresholds are reported in Table 4

The reliability of the classifier based on *y*_*opt*_ can be inferred by considering the confusion matrix of Table 5 (see also the scatter plot in Fig. 4). The accuracy is 94 %, similar to that provided by the classifier relying on *Δ* *x*_205_ only, and equal to that provided by the classifier relying on *y*_*DV*_. However, if only high-reliability responses are considered, namely those with odds at least 90:10, the accuracy of the classifier based on *y*_*opt*_ is still 91 %, slightly better than 88 % provided by *y*_*DV*_ and definitely larger than 64 % given by the classifier relying on *Δ* *x*_205_ only. The improvement with regard to a classifier based on this last measure is pointed out by the ROC curves that are also shown in Fig. 4.

In the case of the classifier based on *y*_*opt*_, by relying on Eqs. (3, 4) a maximum accuracy of 96.1 *%* ± 2.4 *%* can be predicted. By comparison, this last parameter is 95.6 *%* ± 2.6 *%* in the case of the classifier based on *y*_*DV*_.

### Test of the improved classifier on an independent data set

**Fig. 5:**
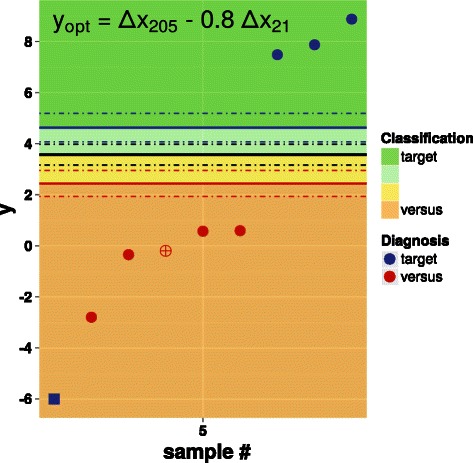
Scatter plot of *y*_*opt*_=*Δ* *x*_205_−0.8·*Δ* *x*_21_ applied to an independent set of data. See Section “A classifier for ADC vs. SQC” and the caption of Fig. 2 for the color code of dots, lines and shaded areas. The values of the thresholds are reported in Table 4. The empty, red dot and the square, blue dot refer to a standard variability outlier and a “bias” outlier, respectively (see main text)

Figure 5 shows the results of the application on a set of 9 additional samples of the classifier based on *y*_*opt*_ and using the population mean, population standard deviation, and thresholds expressed in Tables 3 and 4. With the exception of one single case, all values of the triplicate standard deviations comply with the respective *σ*_max_ requirements explained above. The single outlier is a miR-21 triplicate whose standard deviation of 0.52 slightly exceeds the maximum value of 0.46 (see Table 1) given by the significance level *α*=0.05. Of the remaining 8 samples, the classification provided by the classifier of Eq. (1) coincides with the immunohistochemical diagnosis for 7 samples; in all these cases, the odds are at least 90:10 (the same would happen for the sample containing the miR-21 outlier).

The miRNA classifier of Eq. (1) provides a different diagnosis than the immunohistochemical analysis only for a single sample. Although this sample does not contain any outlier according to the variability of its triplicates, its value of *y*_*opt*_ appears to be extremely low: according to the statistics of *y*_*opt*_ (see Fig. 3), the probability of getting a more extreme value is *p*<5·10^−4^. This hints, rather than to a misclassification of the sample, to a case of “bias” outlier, i.e. to a possibly wrong assessment of the triplicates, as discussed above. For the sake of comparison, for all other 8 samples, as well as for all 33 samples considered in the previous sections, *p*>0.02

## Discussion and conclusion

We introduced a Bayesian classifier that, on the basis of the expression of miR-205, miR-21 and snRNA U6, discriminates samples into two different classes of pulmonary tumors, normally classified by immuno-histochemical approaches: adenocarcinomas and squamous cell carcinomas. The advantage to use miRNAs is due to the ease of their detection and quantification by qRT-PCR, as well as in their extreme specificity. miRNAs are stable molecules well preserved in formalin fixed, paraffin embedded tissues (FFPE) as well as in fresh snap-frozen specimens, unlike larger RNA molecules as messenger RNAs [3].

Our approach is based on a method that employs the quantification of snRNA U6 as a normalizer, miR-21 as a performance enhancer via noise reduction, and miR-205 as a class discriminator. First, we determined that the variance of miRNA quantification triplicates follows a normal chi-square distribution. Thereupon, we designed a procedure to recognize invalid measures (outliers) and remove them from the analysis. The proof that the measures of interest are compatible with normal distributions makes up a crucial step towards the optimization of the Bayesian classifier, the determination of its performance, inclusively of the related uncertainty, and the identification of “bias” outliers. We then proceeded to optimally set our Bayesian classifier and to determine its performance as well as the related uncertainty. Results are displayed in Fig. 2: the classifier based on miR-205 and normalized on snRNA U6 has the best performance.

A main feature of the Bayesian approach described here is the possibility, also in presence of a limited size of the available data sets, of estimating the reliability of a classifier’s performance. This possibility relies on the verification of the normality of the different distributions of interest. Other powerful methods to set up a classifier, such as support vector machines (SVM) and decision trees, though quite versatile to optimize the decisional parameters on a training set, are less suited than a probabilistic approach to provide an immediate quantification of the reliability in the case of application to new sets of data. For example, in the cases discussed above, a SVM approach would likely result in an optimized classifier that also exploits the “apparent” classification capability of *Δ* *x*_21_ and even *x*_U6_ (see, for example, Figs. 1 and 2). However, according to our analysis based on Student’s t, there is no evidence of such capability, so that such a SVM classifier would also possibly have a larger generalization error than a Bayesian classifier of the kind discussed in this paper.

Finally, we provided a method to enhance a classifiers’ performance by exploiting the correlation between the tumor-discriminating miRNA miR-205 and the expression of miR-21, used as a noise reduction factor. The method essentially consists in exploiting the nonzero covariance of two miRNAs, where the first one acts a classifier and the second one is used to abate the variability of the first one. Figure 4 shows the result of an improved classifier, indicating that only 2 samples lay within the uncertainty region, much less than the 12 samples in the case of the non-improved classifier shown in Fig. 2. Results obtained on an independent data set are also satisfactory.

In conclusion, the proposed method introduces a robust tool for determining the cases in which miRNA quantification can be applied in discriminating inter- and intra-tumoral heterogeneity.

## Endnote

^1^ Because ${f_{\alpha,\,\nu,\,\infty } = \chi ^{2}_{\alpha,\,\nu }/\nu }$, an alternative outlier definition relying on the F-test would produce the same result as that discussed in this section.

## Additional file

Additional file 1 **The datasets are available as Supplementary Material.** (TXT 12.8 KB)
